# Protocol: a versatile, inexpensive, high-throughput plant genomic DNA extraction method suitable for genotyping-by-sequencing

**DOI:** 10.1186/s13007-018-0336-1

**Published:** 2018-08-28

**Authors:** Craig B. Anderson, Benjamin K. Franzmayr, Soon Won Hong, Anna C. Larking, Tracey C. van Stijn, Rachel Tan, Roger Moraga, Marty J. Faville, Andrew G. Griffiths

**Affiliations:** 1 AgResearch Grasslands Research Centre, Private Bag 11008, Palmerston North, 4442 New Zealand; 2 Present Address: Slipstream Automation, Bachelor Centre, Module 4, Tennent Drive, Palmerston North, 4442 New Zealand; 3 AgResearch Invermay Agricultural Centre, Puddle Alley, Private Bag 50034, Mosgiel, 9053 New Zealand

**Keywords:** High-throughput, Freeze-dried, Silica gel-dried, Next-generation sequencing, *Trifolium*, *Lolium*, *Medicago*, *Secale*, *Festuca*, *Malus*, *Oryza*, *Arabidopsis*

## Abstract

**Background:**

The recent development of next-generation sequencing DNA marker technologies, such as genotyping-by-sequencing (GBS), generates thousands of informative single nucleotide polymorphism markers in almost any species, regardless of genomic resources. This enables poorly resourced or “orphan” crops/species access to high-density, high-throughput marker platforms which have revolutionised population genetics studies and plant breeding. DNA quality underpins success of GBS methods as the DNA must be amenable to restriction enzyme digestion and sequencing. A barrier to implementing GBS technologies is access to inexpensive, high-throughput extraction methods that yield sequencing-quality genomic DNA (gDNA) from plants. Several high-throughput DNA extraction methods are available, but typically provide low yield or poor quality gDNA, or are costly (US$6–$9/sample) for consumables.

**Results:**

We modified a non-organic solvent protocol to extract microgram quantities (1–13 μg) of sequencing-quality high molecular weight gDNA inexpensively in 96-well plates from either fresh, freeze-dried or silica gel-dried plant tissue. The protocol was effective for several easy and difficult-to-extract forage, crop, horticultural and common model species including *Trifolium*, *Medicago*, *Lolium*, *Secale*, *Festuca*, *Malus*, *Oryza*, and *Arabidopsis*. The extracted DNA was of high molecular weight and digested readily with restriction enzymes. Contrasting with other extraction protocols we assessed, Illumina-based sequencing of GBS libraries developed from this gDNA had very uniform high quality base-calls to the end of sequence reads. Furthermore, DNA extracted using this method has been sequenced successfully with the PacBio long-read platform. The protocol is scalable, readily automated without requirement for fume hoods, requires approximately three hours to process 192 samples (384–576 samples/day), and is inexpensive at US$0.62/sample for consumables.

**Conclusions:**

This versatile, scalable and simple protocol yields high molecular weight genomic DNA suitable for restriction enzyme digestion and next-generation sequencing applications including GBS and long-read sequencing platforms such as PacBio. The low cost, high-throughput, and extraction of high quality gDNA from a range of fresh and dried source plant material makes this method suitable for many sequencing and genotyping applications including large-scale sample screening underpinning breeding programmes.

## Introduction

Next-generation sequencing (NGS) underpins recently developed high-throughput molecular marker technologies, such as genotyping-by-sequencing (GBS). These methodologies can identify thousands of informative single nucleotide polymorphism (SNP) markers cost-effectively and rapidly in almost any species, regardless of genomic resources [1]. DNA quality is paramount for sequencing and restriction enzyme-based GBS platforms, which require high molecular weight genomic DNA (gDNA) largely free of contaminants, such as polysaccharides or phenols [2, 3]. These contaminants can inhibit sequencing quality as well as restriction enzyme activity and subsequent procedures, thereby providing a source of inconsistent or unreliable SNP data, which has significant implications for genotype analysis [4].

DNA extraction, particularly from plants, can be problematic due to cell wall and fibre components that interfere with tissue homogenisation, in addition to contamination from phenols and carbohydrates. While many protocols are available to address these challenges [3, 5–7], most are expensive (US$6–$9/sample) or relatively low-throughput [3, 8]. Furthermore, many plant extraction methods rely on organic solvents or include compounds such as β-mercaptoethanol [3, 5–7] which render protocol automation without a fume hood difficult. Whitlock et al. [2] described a non-organic solvent, high-throughput DNA extraction protocol suitable for PCR-based assays from freeze-dried plant and insect tissue. We have improved and extended this protocol to routinely extract sequencing-quality high molecular weight gDNA from fresh and dried tissue in a high-throughput manner across a wide range of forage, crop, and model species while significantly enhancing cost and time-effectiveness.

### Materials

#### Reagents

- Sodium chloride (www.thermofisher.com, Cat code: BSPSL944.5).
- Tris (www.thermofisher.com, Cat code: AJA180-500 g).
- EDTA (www.thermofisher.com, Cat code: AJA180-500G).
  CAUTION HSNO Classes: 6.1E (oral), 6.3B, 6.4A, 9.1C (fish). Hazard statement: H303 May be harmful if swallowed; H316 Causes mild skin irritation; H319 Causes serious eye irritation; H412 Harmful to aquatic life with long lasting effects.
- Sodium sulphite (www.vwr.com, Cat code: 28130.260).
- Sodium dodecyl sulphate (www.vwr.com, Cat code: VWRC442444H).
  CAUTION HSNO Classes: 6.1C, 6.1D (oral), 6.3B, 6.4A, 9.1D (algal), 9.1D (crustacean), 9.1D (fish), 9.2D, 9.3C. Hazard statement: H302 Harmful if swallowed, H316 Causes mild skin irritation; H319 Causes serious eye irritation; H413 May cause long lasting harmful effects to aquatic life; H423 Harmful to the soil environment; H433 Harmful to terrestrial vertebrates.
- Potassium acetate (www.sigmaaldrich.com, Cat code: P1147-1 kg).
- Acetic acid (www.merckmillipore.com, Cat code: 1000632500).
  CAUTION HSNO Classes: 3.1C, 6.1D, 6.1D (inhalation), 6.1D (oral), 6.9B (inhalation), 8.1A, 8.2B, 8.3A, 9.1D (algal), 9.1D (crustacean), 9.1D (fish), 9.3C. Hazard statement: H226 Flammable liquid and vapour; H290 May be corrosive to metals; H302 Harmful if swallowed; H332 Harmful if inhaled; H314 Causes severe skin burns and eye damage; H318 Causes serious eye damage; H371 May cause damage to organs; H373 May cause damage to organs through prolonged or repeated exposure; H413 May cause long lasting harmful effects to aquatic life; H433 Harmful to terrestrial vertebrates.
- Guanidinium chloride (www.vwr.com, Cat code: ALFAA13543.0B).
  CAUTION HSNO Classes: 6.1C, 6.1D (oral), 8.2C, 8.3A, 9.3C. Hazard statement: H302 Harmful if swallowed; H314 Causes severe skin burns and eye damage; H318 Causes serious eye damage; H433 Harmful to terrestrial vertebrates. Guanidinium chloride is a strong protein denaturing agent.
- Absolute ethanol (www.vwr.com, Cat code: VWRC20821.365).
  CAUTION HSNO Classes: 3.1B, 6.4A. Hazard statement: H225 Highly flammable liquid and vapour H319 Causes serious eye irritation.
- Liquid nitrogen (www.linde-gas.com, Cat code: 4081536554).
  CAUTION Hazard statement: H281 Contains refrigerated gas; may cause cryogenic burns or injury.
- 20 µg µl^−1^ 1000 mg Proteinase K Bioline (www.bioline.com, Cat code: BIO-37039).
- Qiagen RNase A 2.5 ml (100 mg ml^−1^; 7000 units ml^−1^) (www.qiagen.com, Cat code: QIA19101).
- LabServ Pronalys 2–6 mm self-indicating Silica gel (www.thermofisher.com, Cat code: BSPSL519.5).
- 1 Kb Plus Ladder (www.thermofisher.com, Cat code: 10787-018).

#### Equipment

- Corning^®^ 96 Well Clear Round Bottom 1 ml Polypropylene Block (www.corning.com, Cat code: COR3959).
- 5/32 inch 440C Stainless steel balls grinding grade (3.97 mm) (www.glenmills.com, Cat code: 7400-003969-6).
- 4titude Thermal bond (www.4ti.co.uk, Cat code: 4ti-0591).
- Qiagen TissueLyser II (www.qiagen.com).
- Hettich Rotanta 460R centrifuge (www.hettichlab.com).
- Axygen^®^ 1.1 ml 96 well Deep well plate, clear (www.corning.com, Cat code: P-DW-11-C).
- Axygen^®^ Sealing Mats (www.corning.com, Cat code: AM-2ML-RD).
- Pall AcroPrep™ Advance 96-Well 1 ml Filter plate (www.pall.com, Cat code: PN8132).
- 3/32 inch 440C Stainless steel balls grinding grade (2.38 mm) (www.glenmills.com, Cat code: 7400-002381-6).
- 4titude PCR seal (www.4ti.co.uk, Cat code: 4ti-0500).
- 2 ml Sarstedt Screw Cap Microtubes (www.sarstedt.com, Cat code: SARS72.694.005).
- ¼ inch Ceramic Spheres (www.vwr.com, Cat. No. QBIO6540-034).
- Eppendorf^®^ Microcentrifuge 5415D (www.eppendorf.com).
- Hamilton Microlab^®^ STARlet Liquid Handling Workstation (www.hamiltoncompany.com).
- Nanodrop ND-1000 Spectrophotometer (www.thermofisher.com).
- Qubit^®^ 2.0 Fluorometer (www.thermofisher.com).

#### Reagent setup

##### *Homogenisation buffer (HB)*

 500 mM sodium chloride, 100 mM Tris (pH 7.4), 50 mM EDTA, 52 mM sodium sulphite, 0.7% (w/v) sodium dodecyl sulphate. Buffer may be stored for several months at room temperature.

##### *Precipitation buffer (PB)*

 3.6 M potassium acetate, 2.4 M acetic acid. Buffer may be stored for several months at room temperature.

##### *Binding buffer (BB)*

 To make up 1000 ml, add 191 g of 2 M guanidinium chloride, make up to 333 ml with TE (Tris EDTA 10:1, pH 8), add 667 ml absolute ethanol. Buffer may be stored for several months at room temperature.

##### *Wash buffer (WB)*

 To make up 1000 ml, add 4 ml 5 M sodium chloride, 2 ml 1 M Tris–HCL (pH 8), 194 ml water, 800 ml absolute ethanol. Buffer may be stored for several months at room temperature.

##### *Plant material*

 White clover (*Trifolium repens*), western clover (*T. occidentale*), pale clover (*T. pallescens*), red clover (*T. pratense*), subterranean clover (*T. subterraneum*)*, T. uniflorum*, perennial ryegrass (*Lolium perenne*), tall fescue (*Festuca arundinacea*), rye (*Secale cereale*) and lucerne/afalfa (*Medicago sativa*) were sourced from the Margot Forde Germplasm Centre (MFGC), Grasslands Research Centre, AgResearch, Palmerston North, New Zealand. Plants were grown in planting trays or planter bags in glasshouses on site and leaf or grass pseudostem tissue was harvested for DNA isolation. Leaf tissue from apple (*Malus pumila*) was sampled from trees growing on the AgResearch Grasslands Research Centre campus. Rice (*Oryza* sativa) pseudostem and *Arabidopsis thaliana* leaves were sourced from AgResearch colleagues.

### Protocol

#### Fresh tissue gDNA extraction protocol

*Critical step* Matching bead size with the appropriate well size and plate strength is important to ensure effective tissue grinding and reduces the likelihood of beads jamming in the base of the well, or the plate shattering. To upscale gDNA extraction, the plate may be filled with samples from a single individual.

1. Load 50 mg of fresh tissue and two 5/32 inch (3.97 mm) stainless steel beads into each well of a Corning^®^ 1 ml deep well 96-well plate. Heat-seal with a Thermal Bond seal, which provides greater protection against beads breaking through the seal during the grinding process, and float the plate in liquid nitrogen for 5 min. Similarly, pre-chill Qiagen TissueLyser plate adapters.
2. Place the 96-well plate in the Qiagen TissueLyser plate adapter and grind tissue at 30 Hz for 1 min then, for more consistent grinding, repeat after changing the plate orientation in the adaptors. If homogenising only one plate at a time, use a second balance plate.
3. Centrifuge up to 4000×*g* for 1 min at − 15 °C in a Hettich Rotanta 460R centrifuge (or similar) with a swing bucket rotor for plates to settle plant material to the base of the wells.

##### *Pause point*

 If the plates are not going to be processed at this time they can be stored at − 80 °C until processing is resumed.

##### *Critical step*

 Care must be taken not to allow the samples to thaw prior to adding the homogenisation buffer as this has been shown to adversely affect DNA quality, particularly for recalcitrant species such as white clover.

4. Place the plates in an ice water bath for 20 min to warm to approximately 0 °C. Float them in a tin-foil receptacle to prevent ice adhering to the bottom of the plate.
5. Centrifuge at 4000×*g* for 1 min at − 15 °C to settle any distributed powdered plant material to the base of the wells. Carefully remove the seal and add 500 µl of pre-heated (55 °C for 10 min) HB buffer plus 1.8 µl of 20 µg µl^−1^ Proteinase K per well from a reservoir using a multichannel pipette. Dry the top surface of the plate and apply a fresh Thermal Bond heat seal. Mix well by shaking up and down manually (do not use a vortex machine), then centrifuge at 4000×*g* for 10 min at room temperature.
6. Transfer 300 µl of the supernatant into a fresh Axygen^®^ 1.1 ml 96-well plate. Either automate with a liquid handling robot such as the Hamilton Microlab^®^ STARlet or pipette manually.
7. Add 300 µl PB buffer. Apply an Axygen^®^ sealing mat and mix the plates well by inverting and shaking manually for 10-20 s.
8. Incubate the plates in ice water bath for 15 min.
9. Centrifuge at maximum speed for 30 min at room temperature. For a Hettich Rotanta 460R centrifuge, maximum speed is 8595×*g*.

##### *Pause point*

 The plates may be left at Step 9 for up to 72 h at 4 °C with no adverse effects on DNA quality.

10. Place a Pall AcroPrep™ Advance 96-Well 1 ml Filter plate on an Axygen^®^ 1.1 ml 96-well deep well plate and add 600 µl BB buffer, followed by 400 µl supernatant per well. Mix well by pipetting up and down gently 10 times.

##### *Critical step*

 Care is needed to avoid any possible cross-contamination due to overflow as each well is close to maximum volume. Additionally, gentle pipette mixing is required to prevent gDNA shearing.

11. Centrifuge for 2 min at 4000×*g* at room temperature. Discard the flow-through and dry the collection plate surface with a paper towel.

##### *Critical step*

 With some plant species and tissues, such as ryegrass pseudostem, we have observed some clogging of filter membranes, likely due to carbohydrates. This has been reduced by removing an incubation step after addition of HB buffer. In the event that wells are clogged after centrifugation, this can be remedied by gently scraping or tapping the filter membrane with individual pipette tips followed by a repeat centrifugation.

12. Wash with 300 µl per well of BB buffer. Centrifuge at 4000×*g* for 2 min at room temperature.
13. Wash with 300 µl per well of WB buffer. Centrifuge at 4000×*g* for 2 min at room temperature.
14. Wash with 300 µl per well of absolute ethanol. Centrifuge at 4000×*g* for 2 min at room temperature. Discard flow-through.
15. Centrifuge at 4000×*g* for 5 min at room temperature to dry the membrane.
16. Swap to a fresh Axygen^®^ 1.1 ml 96-well deep well plate for collection of the DNA to be eluted from the filter plate.
17. Add 115 µl of 10 mM Tris HCl pH 8 and 0.04 µl 100 mg ml^−1^ RNAse A per well (dispense from a reservoir) and centrifuge at 4000×*g* for 1 min at room temperature. This should yield approximately 100 µl of eluent containing 1–13 µg DNA per well, depending upon the plant species and grinding efficiency. Seal the plates for storage with a 4titude PCR sealing film.
18. The DNA is quantified with a Qubit^®^ 2.0 Fluorometer, and 230–280 nm absorbance scans performed on a Nanodrop ND-1000 Spectrophotometer.

### Timing

For one operator extracting two 96-well plates (192 samples):

Step 1, 1 h 40 min (loading tissue may be done in advance and stored for a week at 4 °C).

Steps 2–4, 30 min.

Step 5, 20 min.

Steps 6 + 7, 30 min.

Step 8, 15 min.

Steps 9 + 10, 1 h.

Steps 11–16, 30 min.

#### Freeze-dried/silica gel-dried tissue protocol

This protocol follows the steps described in the Fresh Tissue gDNA Extraction Protocol with the following changes.

##### *Critical step*

 We have found that when freeze-drying, for best long-term plant tissue archiving and DNA quality, we place recently harvested material in a pre-chilled freeze-drier and apply an effective vacuum as soon as possible. This avoids a pre-freezing stage and the potential for tissue to thaw and cell components to rupture in the initial freeze-drying process, which we have found yields degraded gDNA. If freeze-drying facilities are not readily available, particularly if harvesting material in more remote field conditions, an effective alternative is drying plant tissue samples with silica gel. After 36 h of drying using a ratio of 10 g silica gel (LabServ Pronalys 2–6 mm self-indicating Silica gel) per 1 g fresh weight of plant material, sequencing-quality gDNA can be extracted using our modified protocol. This drying method is suitable for leaf material in small batches or 96-well plates. For both methods, fresh material may be stored in sealed bags for up to a week at 4 °C, rather than liquid nitrogen or dry-ice, prior to drying, with no reduction in DNA quality. Harvesting directly into the 96-well plate, drying, adding stainless steel balls, then sealing and storing in a container with silica gel to reduce humidity, provides an effective way to stockpile sample plates in advance of DNA extraction. Drying samples in the plates also avoids potential cross contamination if transferring previously dried, friable and likely electrostatically charged samples into wells.

1. Harvest 50 mg of fresh leaf directly into each well of a Corning^®^ 1 ml deep well 96-well plate, and freeze-dry or silica gel-dry the entire 96-well plate and contents. If the material has already been dried, add 15 mg of dried tissue to each well. Two smaller (3/32 inch; 2.38 mm) stainless balls per well are used for the homogenisation step as the plant tissue is already brittle and readily ground. Heat seal with a Thermal Bond seal.
2. The next steps are identical to the Fresh tissue protocol except that the Qiagen TissueLyser adapters do not need to be pre-cooled, centrifugations may be performed at room temperature, and Steps 4 (ice bath) and 5 are omitted.

#### Individual tube tissue protocol

This protocol follows the steps described in the Fresh Tissue gDNA Extraction Protocol with the following changes.

##### *Critical step*

 This protocol is effective with fresh and freeze/silica gel-dried plant tissue. It is important to match the bead size with the size/strength of the screw-cap microtube to prevent breakage. For cost-effectiveness and consistent gDNA quality, we use a Pall AcroPrep™ Advance 96-Well 1 ml Filter plate rather than individual silica filter spin columns. We use the same plate repeatedly for multiple individual extractions but use a fresh well for each extraction.

This protocol can be performed using option A (Fresh Tissue), or option B (Freeze/silica gel-dried Tissue).

(A) Using fresh plant tissue fresh material.
  1. Harvest 50 mg of fresh leaf directly into a 2 ml Sarstedt Screw Cap microtube, add two 3.97 mm stainless steel balls, and float the tube in liquid nitrogen for 5 min. Similarly, pre-chill Qiagen TissueLyser tube adapters for microfuge tubes.
  2. Place the tube in a Qiagen TissueLyser with tube adapter and grind tissue at 30 Hz for 1 min. Repeat if grinding is insufficient.
  3. Centrifuge at maximum speed in an Eppendorf^®^ Microcentrifuge 5415D benchtop centrifuge (or similar) with a fixed angle rotor to settle powdered plant material to the base of the tube. The protocol follows the same steps as the Fresh Tissue protocol from step 4 except that at step 6 the supernatant is transferred to a 1.5 ml Eppendorf microfuge tube, and a benchtop microcentrifuge is used until step 10.

##### *Critical step*

 We found that using individual silica spin columns did not produce the required gDNA quality. Consequently, we use a Pall AcroPrep™ Advance 96-Well 1 ml Filter plate. For cost effectiveness, we use the same plate repeatedly for multiple individual extractions but use a fresh well for each extraction.

(B) Using freeze/silica gel-dried tissue.
  1. Either harvest 50 mg of fresh leaf directly into a 2 ml Sarstedt Screw Cap microtube and freeze/silica gel dry as described above, or add 15 mg of previously dried tissue. Add a ¼ inch Ceramic Sphere.
  2. Place the tube in a Qiagen TissueLyser with tube adapter and grind tissue at 30 Hz for 1 min. Repeat if grinding is insufficient.
  3. Centrifuge at maximum speed in an Eppendorf^®^ Microcentrifuge 5415D benchtop centrifuge (or similar) with a fixed angle rotor to settle powdered plant material to the base of the tube. The protocol follows the same steps as the Fresh Tissue protocol from step 6 except that at step 6 the supernatant is transferred to a 1.5 ml Eppendorf microfuge tube, and a benchtop microcentrifuge is used until step 10.

##### *Critical step*

 We found that using individual silica spin columns did not produce the required gDNA quality. Consequently, we use a Pall AcroPrep™ Advance 96-Well 1 ml Filter plate. For cost effectiveness, we use the same plate repeatedly for multiple consecutive individual extractions but use a fresh well for each extraction.

## Results

**Fig. 1:**
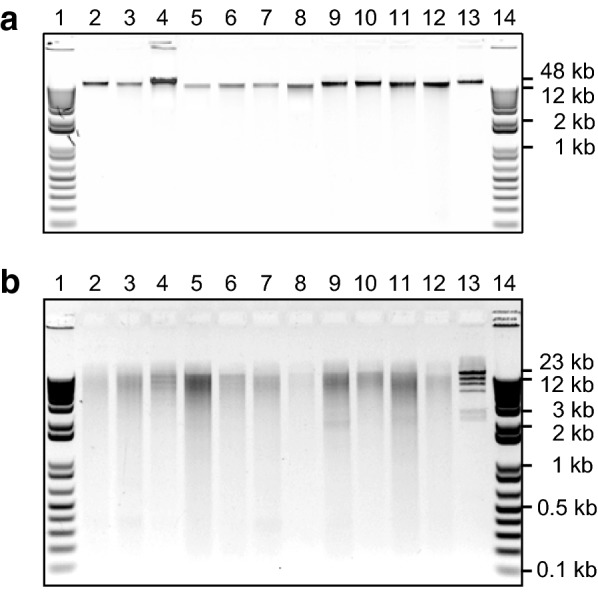
Genomic DNA (gDNA) extracted and restriction endonuclease-digested from forage legumes and grasses, and horticultural species. Samples of gDNA **a** extracted from freeze-dried leaf tissue or grass pseudostem using the high-throughput protocol and **b** digested with *Hin*dIII restriction endonuclease were resolved and visualised by electrophoresis in an agarose lithium borate buffer (0.8% w/v) gel containing 25 μg ethidium bromide. The samples were, in order: (1) 1 kb Plus size ladder (www.thermofisher.com); (2) *Trifolium repens* (white clover); (3) *T. occidentale* (western clover); (4) *T. pallescens* (pale clover); (5) *T. pratense* (red clover); (6) *T. subterraneum* (subterranean clover); (7) *T. uniflorum* (8) *Medicago sativa* (alfalfa/lucerne); (9) *Lolium perenne* (perennial ryegrass); (10) *Festuca arundinacea* (tall fescue); (11) *Secale cereale* (rye); (12) *Malus pumila* (apple); (13) λ DNA standard, and (14) 1 kb Plus size ladder. The λ DNA standard was digested with *Hin*dIII in (**B**)

**Fig. 2:**
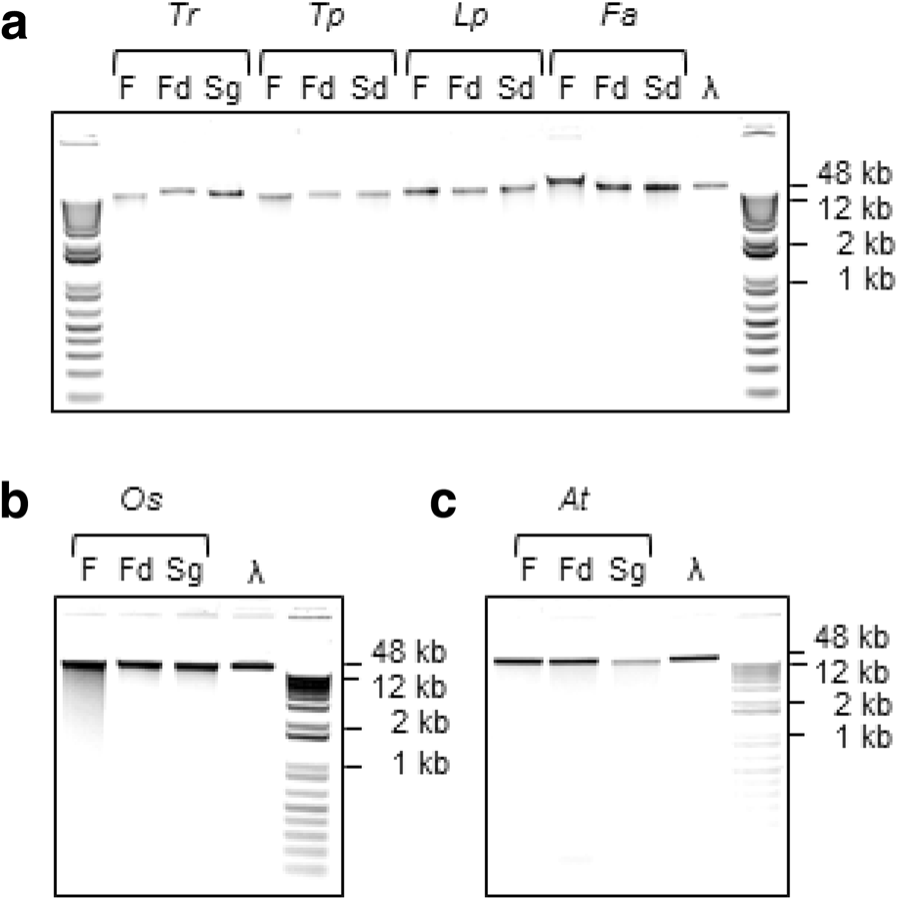
Genomic DNA (gDNA) extracted from fresh, freeze-dried, and silica gel-dried plant tissue. Samples of gDNA extracted from **a** clover leaf tissue or grass pseudostem **b** rice pseudostem and **c** *Arabidopsis thaliana* leaf tissue using the high-throughput protocol were resolved and visualised by electrophoresis in an agarose lithium borate buffer (0.8% w/v) gel containing 25 μg ethidium bromide. *F* fresh, *Fd* freeze-dried, *Sd* silica gel-dried; *Tr Trifolium repens* (white clover); *Tp T. pratense* (red clover); *Lp Lolium perenne* (perennial ryegrass); *Fa Festuca arundinacea* (tall fescue); *Os Oryza sativa* (rice); *At Arabidopsis thaliana*; *λ* λ DNA standard. The samples were flanked by 1 Kb Plus size ladders (www.thermofisher.com)

**Fig. 3:**
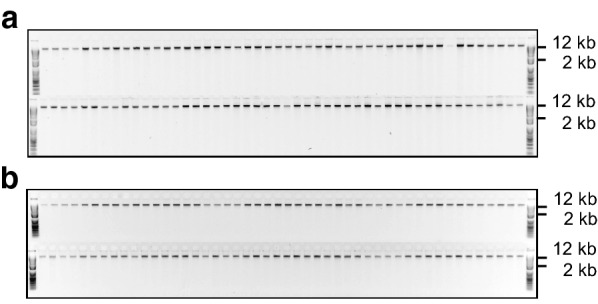
A typical DNA extraction from freeze-dried and fresh tissue using the 96-well plate method. Genomic DNA was extracted from 96 individuals of **a** *Trifolium repens* (white clover; freeze-dried leaf) and **b** *Lolium perenne* (perennial ryegrass; fresh pseudostem) using the 96-well plate protocols, and resolved and visualised by electrophoresis on an agarose lithium borate buffer (0.8% w/v) gel containing 25 μg ethidium bromide. The samples were flanked at either end by 1 Kb Plus size ladders (www.thermofisher.com). One *T. repens* individual had a poor DNA yield (top row, ninth lane from the right) and was subsequently re-extracted using the Individual Tube freeze-dried tissue protocol. These DNA samples have been used for developing genotyping-by-sequencing libraries and yielded high-quality sequence data

Using either fresh or freeze/silica gel-dried source material, this versatile, inexpensive, high-throughput and scalable extraction method produces sequencing-quality genomic DNA (gDNA) from a range of forage and horticultural crops (Table 1). Yields from 50 mg fresh or 15 mg dried material range from 1 to 13 μg (mean = 2.7 μg), depending on the species and tissue, with a mean 260/280 nm absorbance ratio of 2.0 (Table 1). Furthermore, the extracted gDNA is consistently of high molecular weight (Fig. 1a) and is amenable to restriction enzyme digestion (Fig. 1b). Whether extracted from fresh or, where tested, dried plant tissue (Fig. 2), the gDNA is stable at 4 °C for several months although, as with most purified DNA, long-term storage is best at − 20 to − 80 °C. Typically, 96-well plate extractions produce relatively consistent yields across the plate with a low failure rate (Fig. 3), which is important for processing large numbers of individuals with few repeat extractions required.

**Table 1:** Summary of genomic DNA extractions from a range of species and tissue preparations

Species	Tissue^1^	Yield range^2^ (μg)	Yield mean (μg)	A260/280 absorbance ratio	Sequenced^3^
White clover (*Trifolium repens*)	Fresh	1–3	2.5	2.16	GBS
	FD	1–6	2.7	2.17	GBS
	SG	2–6	4.1	1.97	–
Western clover (*T. occidentale*)	Fresh	1.5–13	2.1	2.10	GBS
	FD	1.4	1.4	2.09	GBS
	SG	1.2	1.2	2.09	–
Pale clover (*T. pallescens*)	Fresh	3.5	3.5	2.08	–
	FD	1–6	2.1	1.90	GBS
	SG	2.4	2.4	2.11	–
Red clover (*T. pratense*)	Fresh	1–3	1.5	1.85	GBS
	FD	4.8	4.8	2.12	–
	SG	7.7	7.7	2.11	–
Sub clover (*T. subterraneum*)	Fresh	1.9	1.9	1.87	GBS
	FD	1–6	1.7	2.00	–
	SG	1.8	1.8	2.16	–
*T. uniflorum*	Fresh	2–8	2.3	1.93	GBS
	FD	1.2	1.2	2.10	PacBio
	SG	1.2	1.2	2.12	–
Alfalfa/Lucerne (*Medicago sativa*)	Fresh	1.3	1.3	2.12	–
	FD	2–7	4.0	2.03	GBS
	SG	1.7	1.7	2.04	–
Perennial ryegrass (*Lolium perenne*)	Fresh	2–5	3.4	2.07	GBS
	FD	2.3	2.3	2.04	PacBio
	SG	3.5	3.5	2.07	–
Tall fescue (*Festuca arundinacea*)	Fresh	1–9	3.8	1.91	GBS
	FD	4.4	4.4	2.07	–
	SG	4.5	4.5	2.07	–
Rye (*Secale cereale*)	Fresh	2–7	3.8	1.84	GBS
	FD	–	–	–	–
	SG	–	–	–	–
Apple (*Malus pumila*)	Fresh	1–2	1.2	2.13	–
	FD	1–2	1.1	1.99	–
	SG	1–2	1.4	2.14	–
Rice (*Oryza sativa*)	Fresh	2–4	3.2	2.06	–
	FD	1.4	1.4	2.11	–
	SG	2.3	2.3	2.10	
Thale cress (*Arabidopsis thaliana*)	Fresh	0.6	0.6	2.15	–
	FD	0.5	0.5	2.13	–
	SG	0.2	0.2	2.21	–

^1^*FD* freeze-dried; *SD* silica gel-dried

^2^A single number indicates a single extraction was quantified from that species and tissue

^3^*GBS* used routinely for genotyping-by-sequencing and passes FastQC [9] sequencing quality parameters. *PacBio* used to generate quality PacBio long-read sequencing data

Grasses, cereal and rice samples were pseudostem, whereas all other plant samples were leaf lamina

**Fig. 4:**
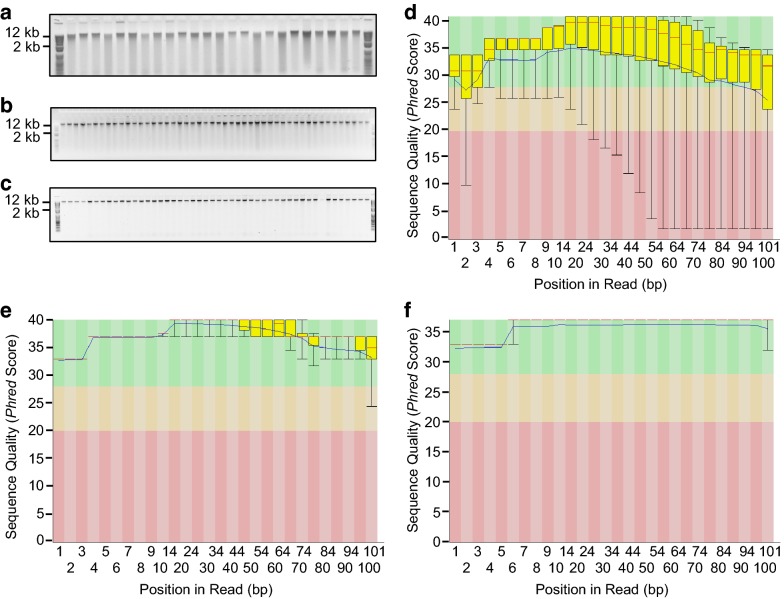
Influence of genomic DNA (gDNA) extraction protocols on sequencing quality. We assessed and modified methodologies to establish a high-throughput protocol to extract gDNA from white clover, a species prone to yielding degraded DNA. Chemistry based on a CTAB protocol [6] was modified for gDNA extraction using 96-well plates (**a**). Adaptation of the Whitlock method [2] to a 96-well plate protocol (**b**), and development of a streamlined inexpensive protocol described in this paper (**c**). In these examples, gDNA was extracted from freeze-dried leaves and aliquots (2 μL from 100 μL gDNA extraction/elution) were resolved and visualised by electrophoresis in an agarose lithium borate buffer (0.8% w/v) gel containing 25 μg ethidium bromide. The samples were flanked at either end by 1 kb Plus size ladders (www.thermofisher.com). Sequence quality of the extracted gDNA was assessed by producing a genotyping-by-sequencing (GBS) library [1] comprising 95 individuals from each of the white clover gDNA extractions shown above (**a**–**c**). The GBS libraries were single-end sequenced (100 bp) on a single lane each of an Illumina 2500 Hi-Seq sequencer. Sequencing quality assessment using FastQC version 0.10.1 [9] for GBS libraries made from the gDNA shown **a**–**c** is represented in graphs describing quality across all bases from every sequence read at each position (**d**–**f**, respectively). Sequence quality is based on *phred* scores [10], an exponential scale where, for example, 20 = one incorrect sequence base-call in 100, and 30 = one incorrect base-call in 1000. The y-axis shows the quality scores, and the higher the score the greater confidence in the base-calls at that position. The background of the graph divides the y-axis into very good quality calls (green), reasonable quality (orange), and poor quality (red)

The extraction protocol influenced the quality of the resulting gDNA and, in turn, the sequencing quality. As we developed the high-throughput methodology for use in a 96-well plate format, we initially used chemistry based on an organic solvent CTAB protocol [6], however this routinely yielded degraded DNA from white clover (Fig. 4a), a recalcitrant species for quality gDNA extraction. The quality of isolated white clover gDNA improved with the Whitlock method [2] (Fig. 4b) which was subsequently modified and streamlined as described in this protocol (Fig. 4c). To assess sequencing quality of the gDNA, a genotyping-by-sequencing (GBS) library [1] made from 95 white clover individuals using the CTAB-extracted gDNA shown (Fig. 4a) was single-end sequenced (100 bp) on a single lane of an Illumina HiSeq 2500 sequencer. Using FastQC version 0.11.5 [9] quality assessment, the data indicated while there were many good *phred* quality [10] sequences among the reads, there were also many poor quality base calls, and sequencing quality decreased further along the 100 bp read length (Fig. 4d). This has consequences for genotyping, as poor quality sequence is a source of inconsistent or unreliable SNP data which has significant implications for genotype analysis [4]. In contrast, GBS libraries made from white clover gDNA extracted adapting the Whitlock [2] method to a 96-well plate (Fig. 4b) yielded good quality sequence, although there was an increase in lesser quality calls in a greater proportion of reads the further along the sequence read (Fig. 4e). White clover gDNA extracted using the streamlined, high-throughput methodology developed in this paper (Fig. 4c) generated GBS libraries that had very uniform high quality base calls to the end of the sequence read (Fig. 4f). This indicates most sequence reads were of similar high quality throughout the lane of data, which provides confidence in downstream genetic analysis. This high-quality sequence output is routine when sequencing gDNA extracted using this protocol across a range of species (Table 1). Furthermore, gDNA extracted from *L. perenne* and *T. uniflorum* using this method was sequenced successfully using the PacBio long-read platform (Macrogen, South Korea) (Table 1).

## Discussion

This modified within-plate DNA extraction protocol has been tested successfully with a wide range of forage, crop, horticultural and model plant species using both fresh and archived freeze-dried/silica gel-dried samples. The sequence quality using gDNA extracted with this protocol, for either genome sequencing via Illumina or PacBio platforms, or reduced-representation genotype-by-sequencing (GBS), is very high. While the buffers and solutions can be made in-house, key changes that have improved cost-effectiveness over previous protocols include substituting pre-packed sets of 96 individually capped microtubes containing tungsten carbide beads, with a standard 96 deep well plate, stainless steel balls and thermal seals. Changing to a plate and thermal seal system saves time and effort at the tissue homogenisation step without compromising well-to-well purity. However, extracting DNA from plant species not listed in Table 1 may require optimizing bead size, type and number, and volume and type of plant tissue to ensure effective homogenization. Further significant time savings and DNA quality enhancements were achieved by eliminating a one hour incubation step that followed mixing ground tissue with homogenisation buffer and, instead, proceeding immediately to centrifugation and transfer of DNA-containing supernatant into the next step. These modifications reduce DNA degradation noticeably and alleviate carbohydrate clogging of the silica filter plate, an issue often associated with extraction from fresh tissue, particularly perennial ryegrass (*Lolium perenne*) pseudostems. Additional modifications include: extracting from fresh material by floating the 96-well plates on liquid nitrogen to freeze samples prior to grinding; and replacing a chaotropic agent for binding DNA to the silica matrix with a guanidinium chloride/ethanol binding buffer. Furthermore, the tissue homogenisation step can be downscaled to smaller sample numbers using individual 2 ml Sarstedt screw-cap microtubes in place of 96-well plates, if required. Large scale extractions from an individual plant can be achieved by filling a 96-well plate with samples from that individual and pooling the eluted gDNA.

Other than the time required to load tissue samples into a 96-well plate (approximately 40 min), a minor shortcoming of this protocol is that approximately 2% of wells may have low DNA yields due to inefficient tissue grinding in those specific wells. This is easily remedied by repeating the extraction for this subset of affected samples using individual tubes.

In summary, this simple protocol extracts high-quality GBS and sequence-ready gDNA inexpensively (US$0.62/sample for consumables) in a scalable high-throughput manner without organic solvents, magnetic beads or other expensive components. Depending on plant and tissue type, a single operator can isolate microgram quantities (1–13 μg) of high quality, high molecular weight gDNA from 192 samples in three hours, or approximately 384–576 samples per day, in a process that can be readily automated. This method is used routinely to extract sequencing-quality gDNA for multiple applications, and is integral to our GBS and molecular breeding projects in a range of grass and legume forage species.

## Conclusion

We have developed an inexpensive, simple, high-throughput plant genomic DNA extraction method based on a 96-well plate format that rapidly yields high molecular weight gDNA that generates high quality sequence data. The protocol is versatile and robust as it can use fresh and dried tissue from a wide range of species, and it can be up or down-scaled according to requirements. The 96-well plate-based grinding and extraction system streamlines the extraction process, which is readily automatable, and as no organic solvents are required, the protocol can be performed without a fume hood and with minimal personal protective equipment. Due to the low cost (US$0.62/sample for consumables), high-throughput, and consistently high-quality sequence data, this protocol is amenable for projects requiring sequence data across multiple platforms, or less demanding PCR-based applications, from large numbers of individuals. In particular, the protocol is suited to molecular breeding and genetic diversity applications underpinned by sequenced-based molecular marker technologies such as genotyping-by-sequencing.
